# A cap-dependent endonuclease inhibitor acts as a potent antiviral agent against La Crosse virus infection

**DOI:** 10.1128/aac.00186-25

**Published:** 2025-07-23

**Authors:** Kei Konishi, Yoshiyuki Taoda, Manabu Igarashi, Takao Shishido, Kazuya Yasuo, William W. Hall, Yasuko Orba, Hirofumi Sawa, Michihito Sasaki, Akihiko Sato

**Affiliations:** 1 Laboratory for Drug Discovery & Disease Research, Shionogi & Co., Ltd. 13350 , Osaka, Japan; 2 Division of Anti-Virus Drug Research, International Institute for Zoonosis Control, Hokkaido University 12810, Sapporo, Japan; 3 Laboratory for Medicinal Chemistry Research, Shionogi & Co., Ltd. 13350 , Osaka, Japan; 4 Division of Global Epidemiology, International Institute for Zoonosis Control, Hokkaido University 12810, Sapporo, Japan; 5 International Collaboration Unit, International Institute for Zoonosis Control, Hokkaido University 12810, Sapporo, Japan; 6 National Virus Reference Laboratory, School of Medicine, University College Dublin 37438, Dublin, Ireland; 7 Global Virus Network, Baltimore, Maryland, USA; 8 Institute for Vaccine Research and Development (HU-IVReD), Hokkaido University 12810, Sapporo, Japan; 9 Division of Molecular Pathobiology, International Institute for Zoonosis Control, Hokkaido University 12810, Sapporo, Japan; 10 One Health Research Center, Hokkaido University 12810, Sapporo, Japan; IrsiCaixa Institut de Recerca de la Sida, Barcelona, Spain

**Keywords:** cap-dependent endonuclease, La Crosse virus, antivirals, bunyaviruses

## Abstract

La Crosse virus (LACV) infection, the causative agent of La Crosse encephalitis, can lead to severe neurological symptoms and sequelae, particularly in children. Despite annual reports of neurologically symptomatic cases, no effective treatment has yet been established. Bunyaviruses, including LACV, utilize a cap-snatching mechanism for transcription, with a cap-dependent endonuclease (CEN) serving as a promising target for antiviral treatment. Specifically, we now demonstrate that a CEN inhibitor, carbamoyl pyridone carboxylic acid (CAPCA)-1, exhibits potent anti-LACV activity *in vitro* and *in vivo*. CAPCA-1 exhibited 50% effective concentration values below 1 µM in neuronal and non-neuronal cells, demonstrating a higher *in vitro* activity than the nucleoside analogs, ribavirin and favipiravir. Multiple passages of LACV in the presence of CAPCA-1 produced numerous amino acid mutations in the CEN active site. Notably, using a lethal infection model in mice, CAPCA-1 treatment reduced viral loads in the brain and extended the survival rate of LACV-infected mice. These findings highlight the potential of CEN inhibitors as treatment options for La Crosse encephalitis.

## INTRODUCTION

La Crosse virus (LACV), an arthropod-borne virus, is classified under the genus *Orthobunyavirus* within the family *Peribunyaviridae*, order *Elliovirales*, and class *Bunyaviricetes* (1). LACV was first isolated from the brain tissue of a 4-year-old girl in 1964 in Wisconsin, USA, and is the primary causative agent of La Crosse encephalitis (2). Numerous cases with neurological symptoms are consistently reported each year, and concerns about the potential for geographic expansion of this arthropod-borne infectious disease are increasing. Despite extensive research on LACV as a representative bunyavirus, no established treatment is currently available (3, 4).

Several small molecule compounds exhibit anti-LACV activity (5–8). Ribavirin (RBV) and T-705 (also known as favipiravir) are nucleoside analogs with broad-spectrum antiviral activity and have been previously evaluated for their anti-LACV activity (9, 10). While RBV has displayed antiviral activity *in vitro*, its efficacy is limited in the *in vivo* infection model, and clinical trials have failed to observe the effectiveness of RBV in the treatment of LACV encephalitis (11, 12). T-705 extends survival in mice infected with the Jamestown Canyon virus and Ebinur Lake virus, both members of the *Orthobunyavirus* genus (13, 14). However, its efficacy against LACV is limited in animal models (15), and its therapeutic potential for orthobunyavirus infections remains uncertain.

Bunyaviruses, including LACV, initiate mRNA transcription through a mechanism termed cap-snatching (16, 17). This process relies on a cap-dependent endonuclease (CEN), a part of L protein encoded by the viral *L* gene, which cleaves several nucleotides downstream of the host mRNA cap structure. The capped RNA fragment is then utilized as a primer for transcription initiation. Among bunyaviruses, the PD (E/D) K motif is highly conserved in the CEN active site, and its enzymatic activity requires divalent cations (18). Humans do not have a CEN homologous gene, making it an ideal virus-selective drug target. Indeed, the CEN inhibitor baloxavir acid (BXA), which is clinically employed for treating influenza virus (IFV) infection, has been optimized as an antiviral agent with chelating divalent cations responsible for the inhibition of IFV CEN activity (19, 20). Furthermore, several studies have reported that BXA binds to the CEN of orthobunyaviruses and inhibits its enzymatic activity (21, 22).

In a previous study, we screened 6,077 two-metal chelating compounds, including CEN inhibitors, and identified four carbamoyl pyridone carboxylic acids (CAPCAs) (previously named compounds A, B, C, and D) as anti-arenavirus agents (23). These four compounds also inhibited the IFV CEN enzyme and viral replication. Bunyaviruses, including LACV, were most susceptible to CAPCA-1 (previously named compound B) among the four CEN inhibitors (23). However, the effects of CAPCA-1 on LACV are preliminary, and its antiviral activity, including reduced viral load and therapeutic efficacy in infected animals, remains poorly understood. Because of the neurotropic nature of LACV (24, 25), evaluation of the antiviral activity of CAPCA-1 in neuronal cells remains necessary. The present study aimed to determine whether the CEN inhibitor, CAPCA-1, is effective against LACV infection and to assess its potential as a treatment option by characterizing its anti-LACV activity using *in vitro* and *in vivo* infection models.

## MATERIALS AND METHODS

### Cells and viruses

The African green monkey kidney cell line Vero (JCRB0111; JCRB, Osaka, Japan) and human neuroblastoma cell line SH-SY5Y (EC94030304; ECACC, Salisbury, England, UK) were maintained in high-glucose Dulbecco’s modified Eagle’s medium containing 10% fetal bovine serum (FBS). LACV (VR-1834; ATCC, Manassas, VA, USA) was propagated in Vero cells. The virus stock was titrated using a plaque assay as detailed below and stored at −80°C until further use.

### Compounds

The cap-dependent endonuclease inhibitors, CAPCAs, were synthesized and supplied by Shionogi & Co., Ltd. T-705 (AG002R9Q; Angene, Nanjing, China), RBV (188-02333; Wako, Osaka, Japan), anti-LACV Gc monoclonal antibody (clone 8C2.2; Thermo Fisher Scientific, Waltham, MA, USA), and a monoclonal antibody for the LACV G1 protein (also known as Gc protein) (VR-1821; ATCC) were commercially available. For the *in vitro* experiments, CAPCAs, T-705, and RBV were dissolved in dimethyl sulfoxide (DMSO). For the *in vivo* experiments, CAPCA-1 was dissolved in 0.5% (wt/vol) methylcellulose containing 5% (vol/vol) DMSO, and T-705 was suspended in 0.5% (wt/vol) methylcellulose.

### Immunofluorescence assays

Vero cells seeded in 24-well plates were infected with LACV at a multiplicity of infection (MOI) of 0.01. After 1 h of incubation, the viral inoculum was removed, and Vero cells were cultured with or without the compounds. LACV-infected Vero cells were fixed with 3.7% formaldehyde in PBS at 32 h post-infection (hpi). Subsequently, the cells were permeabilized in PBS with 0.5% Triton X-100 for 5 min and blocked with PBS with 1% bovine serum albumin for 30 min. After blocking, the cells were incubated with a 1,000-fold diluted LACV monoclonal antibody (clone 8C2.2) for 1 h and washed with PBS three times. Subsequently, the cells were incubated with a 500-fold diluted goat anti-mouse IgG (H + L) highly cross-adsorbed secondary antibody Alexa Fluor Plus 488 (Thermo Fisher Scientific) and Hoechst 33342 (Thermo Fisher Scientific) for 1 h. Fluorescent images were obtained using an IX73 fluorescence microscope (Olympus, Tokyo, Japan).

### Cytopathic effect (CPE)-based antiviral and cell viability assays

CPE-based antiviral activity and cell viability assays were conducted as previously reported (23), with several modifications. Vero and SH-SY5Y cells were treated with serially diluted compounds in minimum essential medium (MEM) containing 2% FBS. In the antiviral evaluation, the cells were infected with LACV (40–80 plaque-forming unit [PFU]/well for Vero and 300–4,000 PFU/well for SH-SY5Y cells). In the cell viability assays, the cells treated with the compounds were cultured in the absence of viral infection. After 2 or 3 days of culture, the cells were stained with 3-(4,5-dimethylthiazol-2-yl)-2,5-diphenyltetrazolium bromide (MTT). The antiviral activity (50% effective concentration values, EC_50_) was calculated using Prism version 9.5.1 (GraphPad, La Jolla, CA, USA) using the four-parameter logistic method. Non-infected and non-treated cells were defined as the 100% inhibition control, while LACV-infected and non-treated cells were defined as the 0% inhibition control. Cell viability (50% cytotoxicity concentration values, CC_50_) was calculated using the MTT assay. Non-treated control cells were defined as the 100% cell viability control, while wells containing culture medium without cells were defined as the 0% cell viability control. Selective Index (SI_50_) was calculated as the ratio of CC_50_ to EC_50_ (CC_50_/EC_50_).

### *In vitro* viral growth assays

The cells seeded in 24-well plates were infected with LACV at an MOI of 0.01 for Vero cells and 0.001 for SH-SY5Y cells. After 1 h of incubation with the viral inoculum at 37°C, the cells were maintained in MEM with 2% FBS, with or without the compounds. Culture supernatants were collected at 32 hpi for Vero cells and 48 hpi for SH-SY5Y cells. For growth kinetics analysis comparing CEN mutants and wild-type (WT) virus, both Vero and SH-SY5Y cells seeded in 24-well plates were infected at an MOI of 0.01. After 1 h incubation at 37°C, the cells were maintained in MEM with 2% FBS. Supernatants were collected at 1, 24, 48, and 72 hpi. The viral titers in the supernatants were determined using plaque assays.

### Plaque assays

Monolayers of Vero cells were inoculated with serially diluted virus stocks, cell supernatants, or tissue homogenates for 1 h at 37°C. The viral inoculated media were then removed, and the cells were overlaid with MEM containing 5% FBS, 0.5% methylcellulose, and 10 µg/mL gentamicin. The cells were fixed using 3.7% formaldehyde in PBS at 3 days post-infection (dpi) and subsequently stained with 1% crystal violet.

### Time-of-addition assays

LACV was preincubated with or without the respective compounds at 37°C for 1 h. A mixture of the virus and each compound, or the virus alone, was subsequently inoculated into Vero cells at an MOI of 0.01. After 1 h of incubation, the cells were washed three times with MEM containing 2% FBS and cultured with or without each compound. Total RNA was extracted from the cells at 8 hpi, and viral RNA expression relative to the endogenous controls (nonhuman primate *Actb*) was evaluated by quantitative RT-PCR employing the ΔΔCt method.

### RNA extraction and quantitative RT-PCR

Total RNA was extracted from Vero cells, culture supernatants, and mouse tissues using the Direct-Zol RNA MiniPrep Kit (Zymo Research, Irvine, CA, USA) following the manufacturer’s protocol. RNA was extracted from the mouse whole blood using a High Pure Viral Nucleic Acid Kit (Roche Diagnostics, Mannheim, Germany). qRT-PCR was performed using a Thunderbird Probe One-step qRT-PCR Kit (Toyobo, Osaka, Japan) and StepOnePlus Real-Time PCR System (Thermo Fisher Scientific). The LACV primers and probe sequences were designed with slight modifications to those previously described (Forward, 5′-TATAAAAGCCTAAGAGCTGCCAGAGT-3′, Reverse, 5′-GACCAGTACCGCAGTAATTATAGACAAT-3′, Probe: 5′-FAM-TGTGCAAGTCGAAAGGGCCTGCA-BHQ1-3′) (26). The primers and probes used for the nonhuman primate *Actb* were as previously described (27).

### Selection of LACV resistant to CAPCA-1

Vero cells seeded in a 100 mm culture dish were infected with LACV at an MOI of 0.01. At 3 dpi, 100 µL of the cell-supernatant mixture was added to Vero cells in 24-well plates treated with twofold serial dilutions of CAPCA-1, starting from 30 µM (passage 1). Vero cells without CAPCA-1 were employed as the passaged control. At 2 dpi from passage 1, 100 µL of the cell-supernatant mixture was passaged to naive Vero cells with CAPCA-1, and the procedure was repeated until a complete CPE was observed in Vero cells treated with 30, 15, or 7.5 µM of CAPCA-1. RNA was extracted from the passaged cell-supernatant mixture lysed in TRIzol LS and then analyzed by next-generation sequencing (NGS) to identify mutation sites in the LACV genome. CAPCA-1 susceptibility was tested using a CPE-based antiviral activity assay with passaged LACV.

### Limiting dilution

Passaged LACV was serially diluted 10^2^- to 10^10^-fold in MEM containing 2% FBS. The diluted virus was inoculated into Vero cells seeded in 96-well plates, and the cells were cultured in the presence of 2.5 µM CAPCA-1. At 3–6 dpi, culture supernatants were collected from the wells showing CPE at the highest dilution. These supernatants were then used to inoculate Vero cells in 24-well plates for virus propagation. On day 2 post-inoculation, viral RNA was extracted from the supernatants and subjected to NGS to confirm that the mutation frequency in the CEN region had reached 100%.

### Genome sequence analysis using NGS

The complete LACV genome was amplified by RT-PCR using a PrimeScript II High Fidelity One-Step RT-PCR Kit (TAKARA, Shiga, Japan) and specific primers (Table S1). RT-PCR products were purified with Wizard SV 96 Binding Plates (Promega). Sequencing libraries were prepared using the Nextera XT DNA Library Preparation Kit (Illumina, San Diego, CA, USA) and sequenced on an iSeq 100 (Illumina). Data analysis was conducted using the CLC Genomics Workbench v21.0.3 software (Qiagen, Hilden, Germany).

### Evaluation of the *in vivo* efficacy of compounds in LACV-infected mice

All animal experiments were approved by the Institutional Animal Care and Use Committee of Hokkaido University (approval no. 24-0078). Five-week-old male BALB/cAJcl mice (CLEA Japan, Tokyo, Japan) were subcutaneously administered CAPCA-1 (3, 10, 30, or 60 mg/kg/day) or orally administered T-705 (60, 200, or 300 mg/kg/day). Four hours after treatment, the mice were infected with LACV intraperitoneally (1.0 × 10^4^ PFU/mouse) or via footpad (8.0 × 10^4^ PFU/mouse). They were then treated with CAPCA-1 once daily (q.d.) or T-705 twice daily (b.i.d.) until 7 dpi. CAPCA-1 was administered into the dorsal region of the mice, with each dose administered to different sites to avoid repeated injections at the same site. The humane endpoints were applied under the following conditions: (i) body weight loss of 20% or more compared to the day of infection, (ii) inability to feed due to hind-limb paralysis or severe seizure. Subcutaneous injections were performed under anesthesia induced by isoflurane.

### Viral RNA and titer determination in LACV-infected mouse tissues

The brains, spleens, and whole blood of LACV-infected mice were collected at 6 and 7 dpi. The brain and spleen were suspended and homogenized in PBS (10% wt/vol). For viral RNA analysis, tissue homogenates were lysed with TRIzol LS (Thermo Fisher Scientific) and subsequently underwent RNA extraction and qRT-PCR. Viral RNA was extracted from the whole blood as above. The viral copy number was quantified using the standard curve method. Primers and probe sequences for LACV were described in the RNA extraction and quantitative RT-PCR section above. For viral titer measurement, the brain homogenates were clarified by centrifugation (1,400 × *g*, 5 min, 25°C) and titrated by plaque assays.

### Binding model of CAPCA-1 with the LACV CEN

A binding model of CAPCA-1 to LACV CEN was constructed based on the crystal structure of LACV CEN in a complex with BXA (PDB ID: 7PLR). Initially, a CAPCA-1 three-dimensional structure was constructed using the LigPrep of the Schrödinger suite 2022-4 (Schrödinger, LLC, New York, NY, USA). Subsequently, the prepared structure of CAPCA-1 was superimposed on that of BXA in 7PLR using the Flexible Alignment program in the Molecular Operating Environment software (version 2022; Chemical Computing Group, Montreal, QC, Canada), and BXA was removed.

### Statistical analyses

All statistical tests were conducted using Prism version 9.5.1. Statistical significance was calculated by one-way analysis of variance (ANOVA) with Dunnett’s post hoc test, one-way ANOVA with Tukey’s test, the log-rank (Mantel–Cox) test, and the Mann–Whitney *U*-test.

## RESULTS

### CAPCA-1 inhibits LACV infection *in vitro*

**Fig 1:**
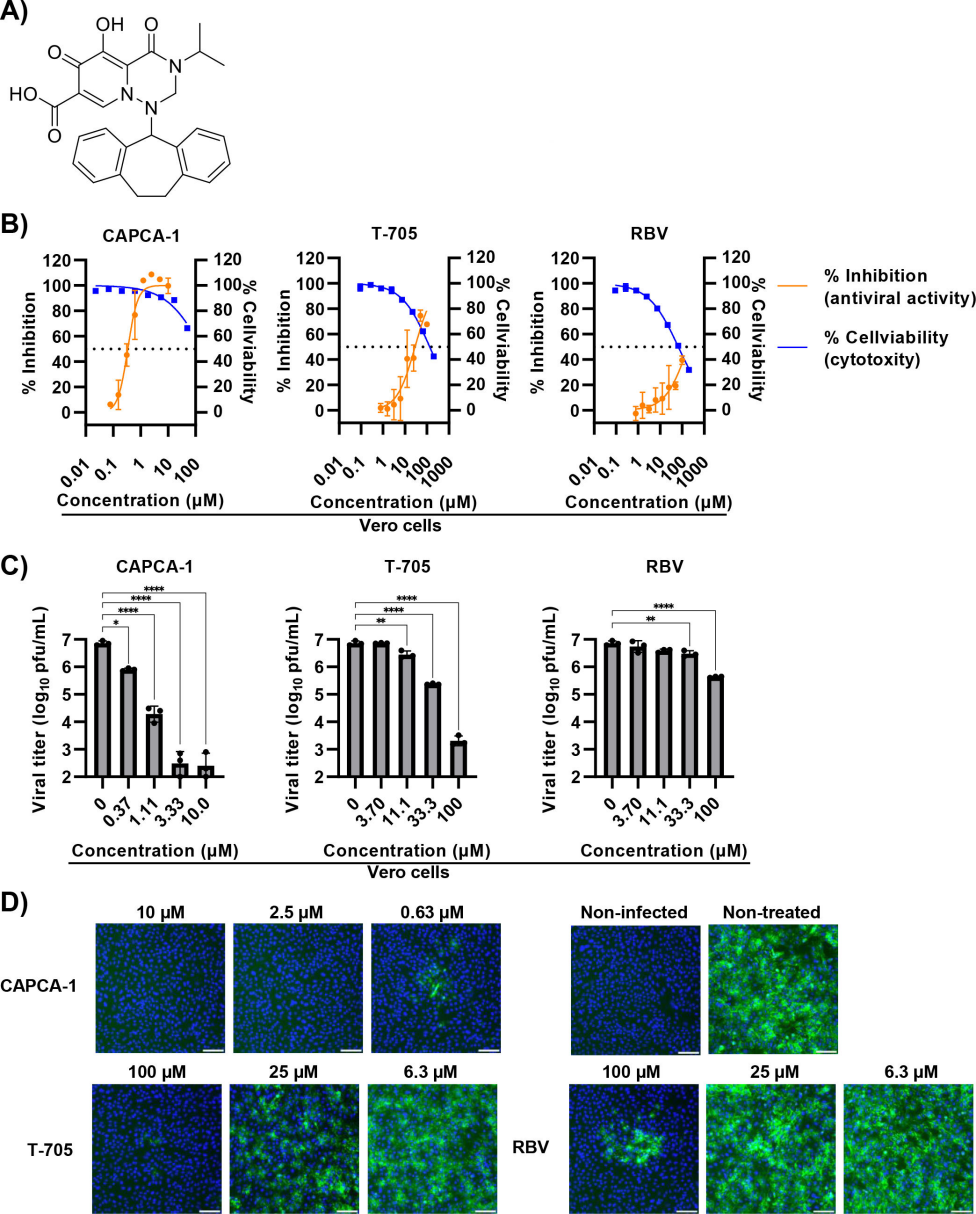
Anti-LACV activity of CAPCA-1 in Vero cells. (**A**) Chemical structure of CAPCA-1. (**B**) CPE-based antiviral activity assays and cell viability in Vero cells. The cells were treated with serially diluted compounds and infected with or without LACV. The inhibition rate and cytotoxicity were assessed using an MTT assay at 3 dpi as described in the Materials and Methods section. These data are representative of three independent experiments performed in duplicate. The dotted lines indicate 50% inhibition and 50% cytotoxicity. Orange and blue lines indicate antiviral activity and cytotoxicity, respectively. (**C**) Viral titer reduction assay in Vero cells. The cells were infected with LACV at an MOI of 0.01. Infected cells were subsequently treated with CAPCA-1 (left), T-705 (middle), or RBV (right). Viral titers in the supernatants were quantified using a plaque assay at 32 hpi. These data are representative of two independent experiments performed in triplicate. (**D**) Images of fluorescent immunostaining. Vero cells infected with LACV at an MOI of 0.01 were treated with CAPCA-1, T-705, or RBV. At 32 hpi, the cells were fixed and stained with an anti-LACV Gc protein antibody (green) and Hoechst 33342 nuclear dye (blue). Scale bars, 100 µm. Data are represented as the mean ± standard deviation (SD) (**B, C**). Statistical analysis was performed by one-way ANOVA with Dunnett’s post hoc test (**C**). *P < 0.05, **P < 0.01, and ****P < 0.0001.

First, we examined the anti-LACV activity of CAPCA-1 compared with the nucleoside analogs (T-705 and RBV) using Vero cells which are widely used for the evaluation of anti-LACV activity (Fig. 1A) (7, 10, 15). Using CPE-based assays, CAPCA-1 inhibited LACV-induced CPE in a noncytotoxic range in a dose-dependent manner, with an EC_50_ value of 0.45 µM. This value was more than 50-fold lower than for T-705 and RBV, with EC_50_ values of 33.85 and >100 µM, respectively (Fig. 1B; Table 1). We further evaluated the anti-LACV activity of CAPCA-1 compared to previously identified CEN inhibitors, CAPCA-2, -3, -4, and -5 in Vero cells (Fig. S1A) (23). Consistent with previous studies (23), CAPCA-1 inhibited LACV-induced cell death at lower concentrations than the other CEN inhibitors (Fig. S1B).

**TABLE 1:** Antiviral activity (EC_50_, µM), cytotoxicity (CC_50_, µM), and SI_50_ of CAPCA-1, T-705, and RBV against LACV^*a*^

	CAPCA-1			T-705			RBV		
	EC_50_	CC_50_	SI_50_	EC_50_	CC_50_	SI_50_	EC_50_	CC_50_	SI_50_
Vero	0.45 ± 0.17	>50	>112	33.85 ± 5.29	117.38 ± 24.66	4.76	>100	82.06 ± 28.4	n.d.
SH-SY5Y	0.69 ± 0.24	>50	>73.2	>100	>200	n.d.	>100	120.97 ± 7.61	n.d.

^*a*^ EC_50_ (µM) is the concentration of antiviral compound that inhibited LACV-induced CPE by 50% relative to the non-treated control. CC_50_ (µM) is the concentration that reduced cell viability by 50% relative to the non-treated control. SI_50_ is the ratio of CC_50_ to EC_50_ (CC_50_/EC_50_). n.d., not determined. Data are represented as the mean ± SD.

We then investigated the effect of CAPCA-1 on viral growth by plaque assays. In Vero cells treated with CAPCA-1, a dose-dependent reduction in infectious virus titers in the culture supernatant was observed, and this was more effective than T-705 or RBV (Fig. 1C). We then attempted to detect LACV Gc proteins using immunofluorescence assays. CAPCA-1 treatment reduced the number of LACV Gc protein-positive cells at lower concentrations compared to treatment with either T-705 or RBV (Fig. 1D).

**Fig 2:**
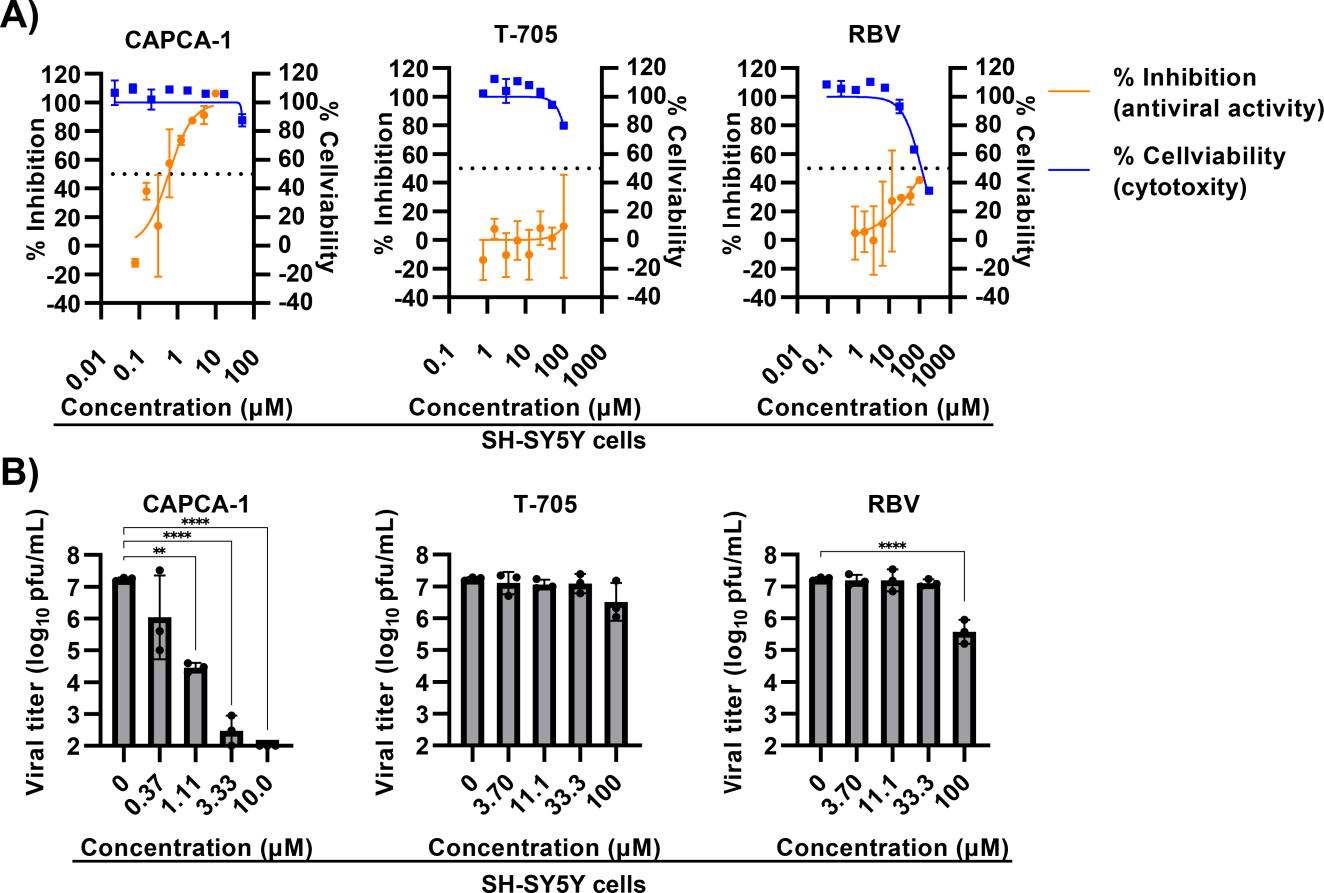
Anti-LACV activity of CAPCA-1 in human neuronal cell lines. (**A**) CPE-based antiviral activity assay and cell viability in SH-SY5Y cells. The cells were treated with serially diluted compounds and infected with or without LACV. The inhibition rate and cytotoxicity were assessed using an MTT assay at 2 and 3 dpi, respectively. These data are representative of three independent experiments performed in duplicate. The dotted lines indicate 50% inhibition and 50% cytotoxicity. Orange and blue lines indicate antiviral activity and cytotoxicity, respectively. (**B**) Viral titer reduction assay in SH-SY5Y cells. The cells were infected with LACV at an MOI of 0.001. Infected cells were subsequently treated with CAPCA-1 (left), T-705 (middle), or RBV (right). Viral titers in the supernatants were quantified using a plaque assay at 48 hpi. These data are representative of two independent experiments performed in triplicate. Data are represented as the mean ± SD (**A, B**). Statistical analysis was performed by one-way ANOVA with Dunnett’s post hoc test (**B**). **P < 0.01 and ****P < 0.0001.

As LACV is detected in neuronal cells in La Crosse encephalitis patients (2, 28, 29), we next investigated the anti-LACV activity of CAPCA-1 in the human neuronal cell line SH-SY5Y. CAPCA-1 reduced the LACV-induced CPE and viral growth in SH-SY5Y, demonstrating potent antiviral activity, with an EC_50_ value of 0.69 µM. In contrast, the EC_50_ values of the nucleoside analogs exceeded 100 µM (Fig. 2A and B; Table 1). This reduced efficacy of nucleoside analogs could be attributed to the inadequate formation of their active forms in neuronal cells (30). CAPCA-1 exhibited antiviral activity comparable to that of CAPCA-4 and demonstrated higher activity than CAPCA-2, -3, and -5 in neuronal cells (Fig. S1C). These results indicate that CAPCA-1 inhibits LACV infection in both non-neuronal and neuronal cells with a higher antiviral activity compared to T-705 and RBV.

### CAPCA-1 disrupts the post-entry phase of LACV infection

**Fig 3:**
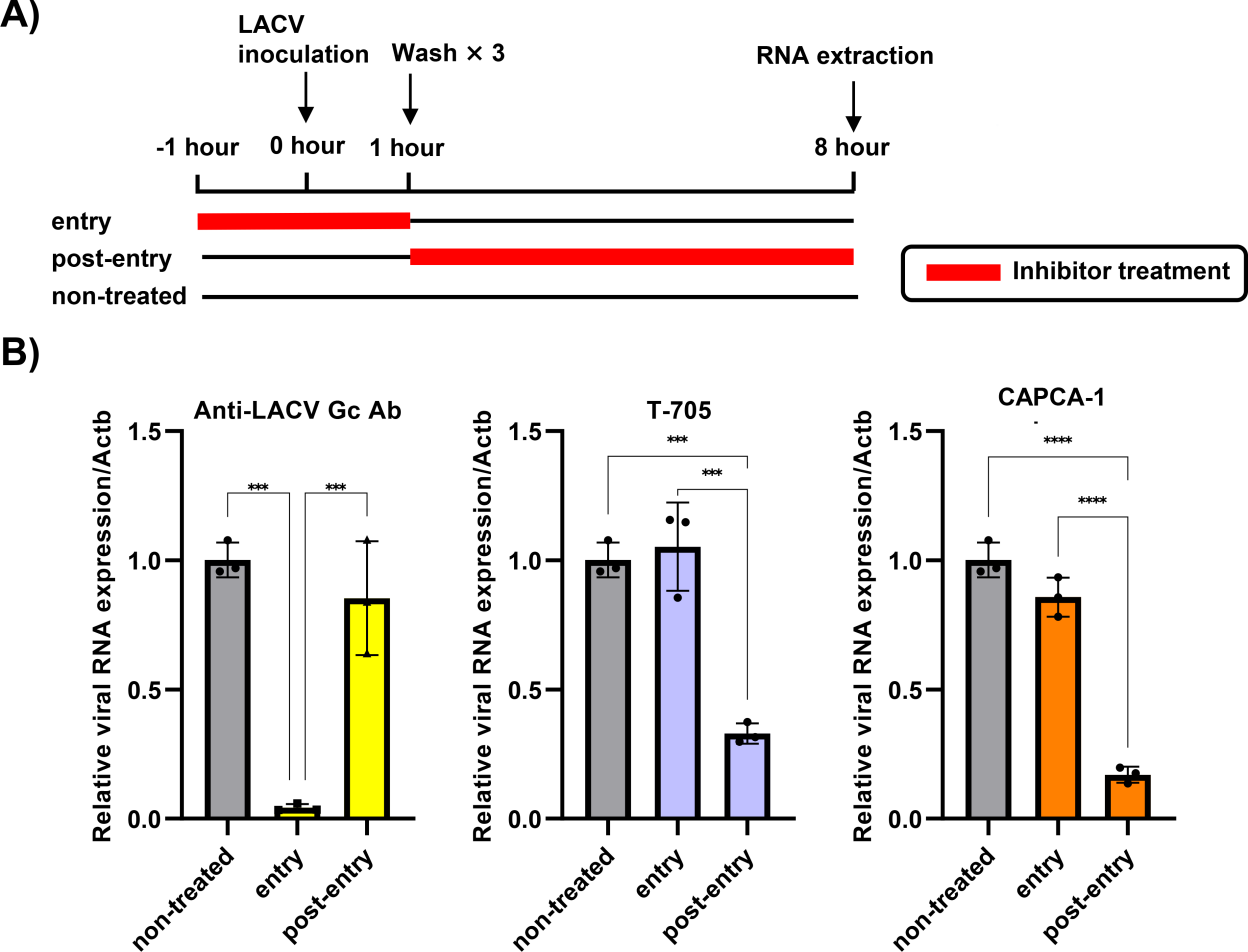
Time-of-addition assay for CAPCA-1. (**A**) Schematic representation of the time-of-addition assay. The virus inoculum was incubated with or without inhibitors for 1 h. Under entry conditions, the virus-inhibitor mixture was added to Vero cells, followed by culture without the compounds. In the post-entry conditions, Vero cells were infected with the virus alone for 1 h, after which the cells were cultured with the inhibitors. Intracellular RNA was extracted at 8 hpi, and viral RNA expression was analyzed by qRT-PCR. (**B**) Viral RNA expression relative to nonhuman primate *Actb* in LACV-infected Vero cells treated with 3 µg/mL of anti-LACV Gc protein monoclonal antibody (left), 200 µM of T-705 (middle), or 10 µM of CAPCA-1 (right). These data are representative of two independent experiments performed in triplicate. Data are represented as the mean ± SD. Statistical analysis was performed using one-way ANOVA with Tukey’s test. ***P < 0.001 and ****P < 0.0001.

We conducted a time-of-addition assay to determine the LACV life cycle stage targeted by CAPCA-1. T-705 and anti-LACV Gc protein monoclonal antibodies were used as assay controls for the inhibitors of replication and entry, respectively. In the entry-stage treatment protocol (Fig. 3A), the anti-LACV Gc antibody (3 µg/mL) significantly decreased viral RNA expression, whereas CAPCA-1 and T-705 had no inhibitory effect (Fig. 3B). In the post-entry treatment protocol (Fig. 3A), CAPCA-1 (10 µM) and T-705 (200 µM), but not the anti-LACV Gc antibody, significantly reduced the viral RNA levels (Fig. 3B). These results indicate that CAPCA-1 targets a post-entry phase of the LACV life cycle, consistent with its character as a viral CEN inhibitor.

### Accumulation of CEN mutations under passage of LACV with CAPCA-1

**Fig 4:**
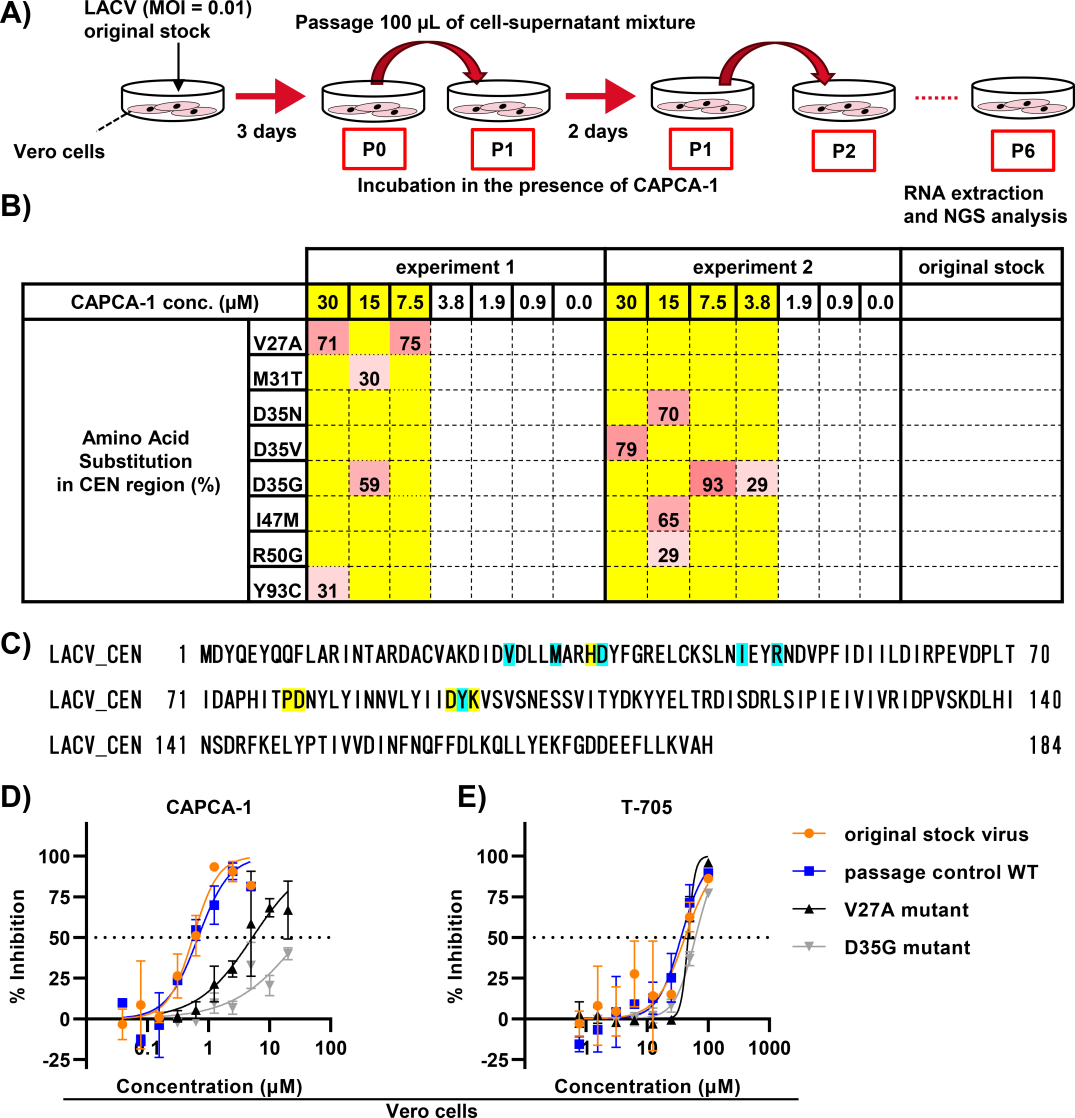
Selection of CAPCA-1-resistant LACV. (**A**) Schematic of the experimental design of selection for CAPCA-1-resistant LACV. LACV was propagated in Vero cells, and the supernatant-cell mixture was added to Vero cells treated with CAPCA-1 (passage 1). LACV was passaged in Vero cells treated with CAPCA-1 every 2 days up to passage 6, after which RNA from the supernatant was extracted for analysis of viral genome mutations using NGS. (**B**) Amino acid substitutions observed at each compound concentration after passage 6 in two independent experiments. The amino acids listed in the table represent substitutions with a mutation frequency of 20% or higher. The yellow highlight indicates the compound concentration at which mutations were observed in the CEN region. The dark pink color represents a mutation rate of 50% or more, while the light pink color indicates a mutation rate of less than 50%. (**C**) Amino acid alignments of the LACV CEN domein (NC_077810). Light blue boxes indicate amino acid substitutions identified in the resistant virus selection assays. Yellow boxes indicate the H....D...PD....DxK motif. The amino acid sequence was created using the Parallel Editor. (**D, E**) CPE-based antiviral activity assay. Vero cells were treated with CAPCA-1 (**D**) or T-705 (**E**) and infected with LACV. The inhibition rate was assessed using an MTT assay. The dotted lines indicate 50% inhibition. Data are represented as the mean ± SD. The original stock virus, passage control WT, V27A mutant, and D35G mutant are represented by orange, blue, black, and gray lines, respectively. These data are representative of more than three independent experiments performed in duplicate.

We attempted to isolate CAPCA-1-resistant LACV to better understand the antiviral mechanisms of this compound. LACV was passaged with twofold serial dilutions of CAPCA-1, starting at 30 µM every 2 days. After six passages, viral RNA was extracted from the cells and supernatant mixtures and subjected to whole-genome sequencing by NGS (Fig. 4A). Several amino acid mutations were identified near the PD(E/D)K motif in the CEN active site of LACV passaged with CAPCA-1 (Fig. 4B and C). The M31T substitution was previously identified in LACV resistant to CAPCA-2 (23) (Fig. 4B and C). Notably, the substitution on Asp35 was reproducibly observed in two independent experiments (Fig. 4B). These mutations were not observed in LACV passaged at 0, 0.9, and 1.9 µM of CAPCA-1 (Fig. 4B). To evaluate the susceptibility of CEN mutants to CAPCA-1, we isolated LACV carrying amino acid substitutions in the CEN region by limiting dilution of passaged virus populations. From LACV passage 6 (P6) at 7.5 µM (with the V27A substitution), 15 µM (with the M31T and D35G substitutions), and 30 µM (with the V27A and Y93C substitutions) in experiment 1, we successfully obtained isolates harboring the V27A or D35G mutations (Table S2). As WT controls, we used both the passage control WT (LACV P6 at 0 µM in experiment 1 was propagated in Vero cells) and the original stock virus (passage 0) (Table S2). In the CPE-based assay using Vero cells, the V27A mutant exhibited a 12.91- and 7.03-fold reduction in CAPCA-1 susceptibility compared to the original stock virus and the passage control WT, respectively (Fig. 4D; Table 2). The D35G mutant showed a >46.51-fold and >25.3-fold reduction in CAPCA-1 susceptibility (Fig. 4D; Table 2). In contrast, all tested viruses exhibited similar susceptibility to T-705 with changes ranging from 0.86 to 1.51 (Fig. 4E; Table 2). Because these viruses were isolated in Vero cells, we further evaluated their susceptibility to CAPCA-1 in SH-SY5Y cells to rule out the possibility that the reduced susceptibility to CAPCA-1 was specific to Vero cells. In the CPE-based assay, the EC_50_ values of RBV and T-705 could not be determined in SH-SY5Y cells (Fig. 2A); therefore, an anti-LACV Gc antibody was used as a control inhibitor. As a result, the V27A mutant exhibited a 4.87-fold increase in EC_50_ values compared to the original stock virus and a 7.12-fold increase compared to the passage control WT (Fig. S2A; Table S3). The D35G mutant showed a 13.57-fold and 19.83-fold increase in EC_50_ values compared to the original stock and passage control WT, respectively (Fig. S2A; Table S3). The efficacy of the antibody remained consistent across all viruses (Fig. S2B; Table S3). These results indicate that the CEN mutants exhibited reduced CAPCA-1 susceptibility not only in Vero cells but also in SH-SY5Y cells. These results support the role of CAPCA-1 as an antiviral agent targeting the LACV CEN.

To assess the replicative capacity of the CEN mutants, we evaluated viral growth kinetics by measuring viral titers in the supernatants of LACV-infected Vero and SH-SY5Y cells. In Vero cells, the viral titers of the CEN mutants were higher than those of the original stock virus and comparable to the passage control WT at 24 hpi. However, by 48 and 72 hpi, titers were similar across all groups (Fig. S2C). This transient increase may reflect adaptation to Vero cells, as both the CEN mutants and the passage control WT had undergone multiple passages in Vero cells. In SH-SY5Y cells, the viral titers of the CEN mutants tended to be lower than those of both the original stock virus and passage control WT (Fig. S2D). These results suggested that the replicative capacity of these CEN mutants exhibited slightly enhanced activity in Vero cells but somewhat impaired in SH-SY5Y cells.

**TABLE 2:** Antiviral activity of CAPCA-1 or T-705 against the original stock virus, passage control WT, and the V27A and D35G mutants in Vero cells^*a*^

	Original stock virus	Passage control WT	V27A mutant	D35G mutant
CAPCA-1 EC_50_ (µM)	0.43 ± 0.10	0.79 ± 0.39	5.55 ± 1.64	>20
Fold change (/original stock)	1.00	1.84	12.91	>46.51
Fold change (/passage control)	0.54	1.00	7.03	>25.32
T-705 EC_50_ (µM)	37.79 ± 3.96	43.84 ± 11.51	44.31 ± 5.15	56.99 ± 11.84
Fold change (/original stock)	1.00	1.16	1.17	1.51
Fold change (/passage control)	0.86	1.00	1.01	1.30

^*a*^ EC_50_ (µM) is the concentration of the antiviral compound that inhibited LACV-induced CPE by 50% relative to the non-treated control. The fold change refers to the ratio of the EC_50_ value for each virus to that of the original stock virus or passage control WT. Data are represented as the mean ± SD.

### CAPCA-1 exhibits anti-LACV activity *in vivo*

**Fig 5:**
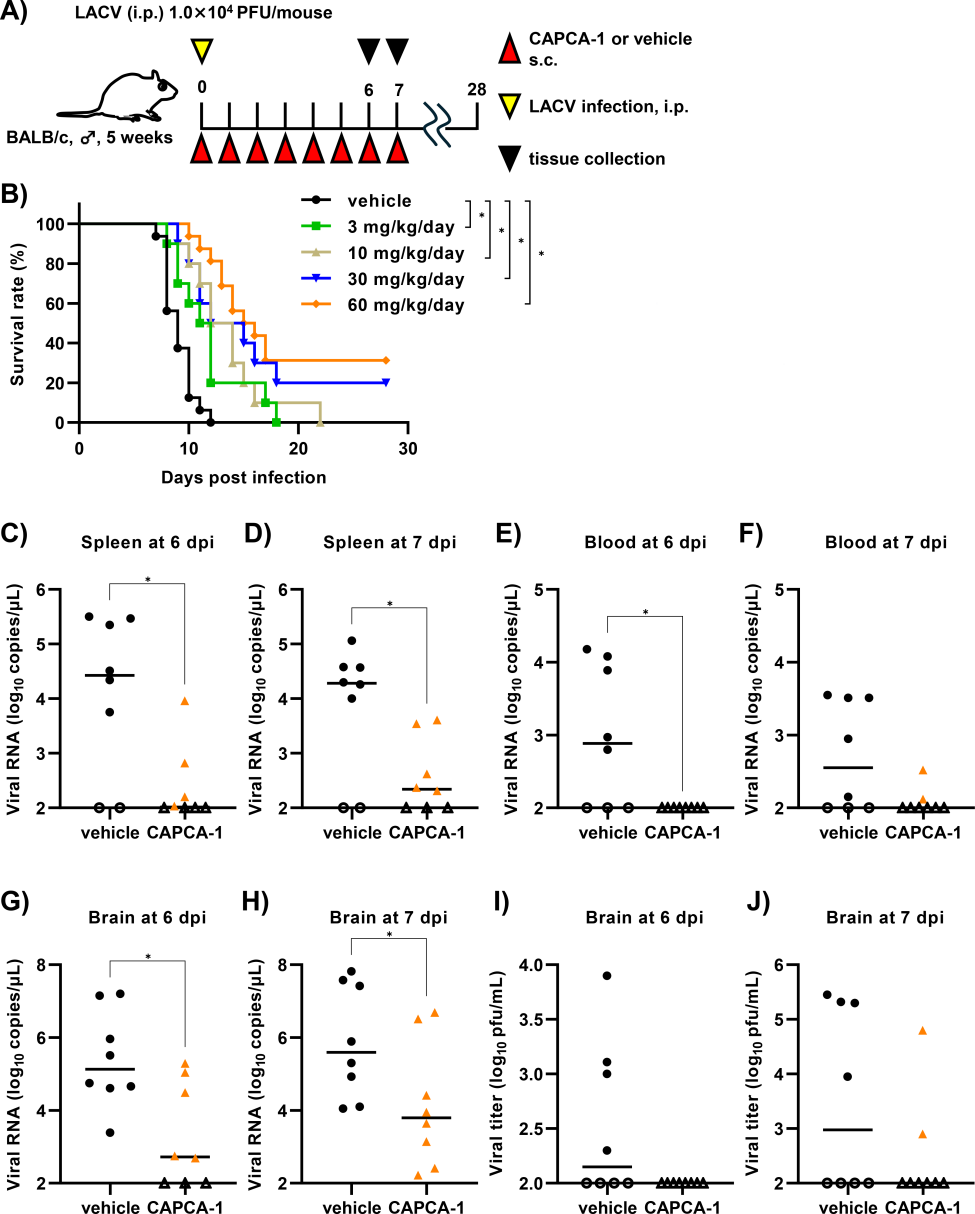
*In vivo* antiviral effect of CAPCA-1 in the LACV-infected mouse model. (**A**) Schematic representation of the experimental design of the prophylactic administration model. Mice were administered vehicle or CAPCA-1 4 h before infection and subsequently intraperitoneally infected with LACV (1.0 × 10^4^ PFU/mouse). Treatment was continued until 7 dpi (q.d., 3, 10, 30, or 60 mg/kg/day). i.p., intraperitoneally; s.c., subcutaneously. (**B**) Survival rates in LACV-infected mice treated with the vehicle (*n* = 16) or CAPCA-1 (3, 10, and 30 mg/kg/day; *n* = 10, 60 mg/kg/day; *n* = 16). (C–H) Viral RNA in the spleen (**C, D**), blood (**E, F**), and brain (**G, H**) in LACV-infected mice treated with vehicle or CAPCA-1 (60 mg/kg/day) at each time point. (**I, J**) Viral titers in the brain in LACV-infected mice treated with vehicle or CAPCA-1 (60 mg/kg) at each time point. Each dot indicates individual mice (C–J). The white circle or triangle indicates below the lower limit of quantification. The bar indicates the median. Statistical analysis was performed by the log-rank (Mantel–Cox) test (**B**) or Mann–Whitney *U*-test (C–J, *P < 0.05 and **P < 0.01). *P* values from the survival analysis were adjusted using the Bonferroni correction, and a *P* value of less than 0.0125 (0.05/4) was considered statistically significant.

Finally, we investigated the anti-LACV effect of CAPCA-1 using a lethal infection mouse model. To evaluate the toxicity of CAPCA-1, uninfected mice were subcutaneously administered CAPCA-1 or vehicle once daily for 7 days. No significant differences in body weight changes were observed between CAPCA-1-treated and vehicle-treated mice throughout the observation period, suggesting that severe toxicity did not occur (Fig. S3A). To evaluate *in vivo* CAPCA-1 efficacy, CAPCA-1 was administered subcutaneously to mice with different doses 4 h before LACV infection, and the mice were then infected with LACV intraperitoneally. CAPCA-1 treatment was continued q.d. until 7 dpi (Fig. 5A). In the survival analysis, vehicle-treated mice succumbed to the LACV infection from 7 to 12 dpi (Fig. 5B). Treatment with 3, 10, 30, or 60 mg/kg/day of CAPCA-1 significantly extended the survival of LACV-infected mice. Treatment with 30 or 60 mg/kg/day rescued 20% and 31% of the mice from lethal infection, respectively (Fig. 5B). The 60 mg/kg/day treatment significantly reduced viral RNA levels in the spleen at 6 and 7 dpi and in the blood at 6 dpi, while a non-significant reduction in viral RNA levels was observed in the blood at 7 dpi (Fig. 5C through F). CAPCA-1-treated mice exhibited significantly lower levels of viral RNA in the brain than the vehicle-treated mice (Fig. 5G and H). Viral titers in CAPCA-1-treated mice were lower, although not significantly, than those in vehicle-treated mice (Fig. 5I and J). We also evaluated the *in vivo* efficacy of T-705 (Fig. S4A). Previous studies have reported the use of 200–300 mg/kg/day as high-dose levels of T-705 (13–15, 31). Based on these reports, 200 and 300 mg/kg/day were selected as the high doses in our *in vivo* efficacy study of T-705. For the low-dose group, we used 60 mg/kg/day, corresponding to one-fifth of the 300 mg/kg/day dose. The prophylactic administration of T-705 in LACV-infected mice did not significantly increase survival rates compared with the vehicle treatment (Fig. S4B).

To assess whether CAPCA-1 inhibits LACV dissemination into the central nervous system (CNS), we evaluated its *in vivo* efficacy using a footpad infection model (32) (Fig. S5A). In this model, viral RNA in the blood was nearly undetectable but tended to be lower in CAPCA-1-treated mice compared to vehicle-treated controls (Fig. S5B). Notably, CAPCA-1 treatment resulted in a significant reduction of viral RNA levels in both the spleen and brain (Fig. S5B), suggesting that CAPCA-1 may suppress LACV dissemination to the CNS. Collectively, these findings indicate that CAPCA-1 reduces the LACV burden and protects mice from lethal LACV infection.

## DISCUSSION

La Crosse encephalitis, a California serogroup virus disease, is caused by LACV infection and can induce severe neurological symptoms in patients. Currently, effective treatments are not available, highlighting an urgent need to develop novel therapeutics. Although the effectiveness of CEN inhibitors against IFV and arenaviruses has been well-characterized, their activity against LACV infections remains unclear. In this study, the CEN inhibitor CAPCA-1 represented more effective anti-LACV activity in viral titers, viral protein expression, and viral-induced cytotoxicity compared with nucleoside analogs. Subcutaneous administration of CAPCA-1 reduced viral loads in the brain and improved survival rates in LACV-infected mice. This study is the first to demonstrate that a CEN inhibitor inhibits LACV infection *in vitro* and *in vivo*, highlighting the potential of CEN inhibitors as antiviral agents against LACV.

Nucleoside analogs, such as T-705 and RBV, require intracellular conversion to their active forms, and their antiviral efficacy depends on the efficiency of this conversion in different cell types (30). Consistent with a previous report (15), our experiments also demonstrated reduced antiviral activity of T-705 in SH-SY5Y cells compared with that in Vero cells. On the other hand, RBV significantly reduced viral titers in both Vero and SH-SY5Y cells in the viral titer reduction assay (Fig. 1C and 2B). Previous studies have reported that RBV inhibits Zika virus replication in SH-SY5Y cells (33), suggesting that, unlike T-705, RBV may be more efficiently metabolized in SH-SY5Y cells. CAPCA-1 effectively reduced viral loads and LACV-induced cell death in both Vero and SH-SY5Y cells, exhibiting EC_50_ values below 1 µM (Fig. 1B, C, 2A and B). Neurons are highly susceptible to LACV infection; LACV-infected neurons undergo apoptosis, which may contribute to neurological damage and subsequent clinical sequelae (34, 35). Therefore, evaluating the antiviral activity in neuronal cells is critical in developing effective treatments for La Crosse encephalitis. Overall, these findings highlight CEN inhibitors as promising antiviral agents with potent activity in neuronal cells.

Mice treated with CAPCA-1 exhibited a significant survival rate and reduced viral loads in the brain compared to vehicle-treated mice (Fig. 5). In our experiments, administration of T-705 did not improve the survival rate of LACV-infected mice compared to vehicle treatment (Fig. S4). Previous research has demonstrated that molnupiravir, a nucleoside analog, and rottlerin administration decreased the incidence of neurological disease in LACV-infected mice (6, 15). To our knowledge, CAPCA-1 is the third compound to show *in vivo* efficacy in a LACV infection mouse model. Prodrug modification is a well-established strategy to enhance oral absorption and improve drug delivery to target organs (36). For instance, baloxavir marboxil is a prodrug of the IFV CEN inhibitor BXA and achieves enhanced oral absorption through modifications of its chelating moiety (37, 38). Given the structural similarity, CAPCA-1 could be modified into a prodrug, potentially allowing oral administration.

**Fig 6:**
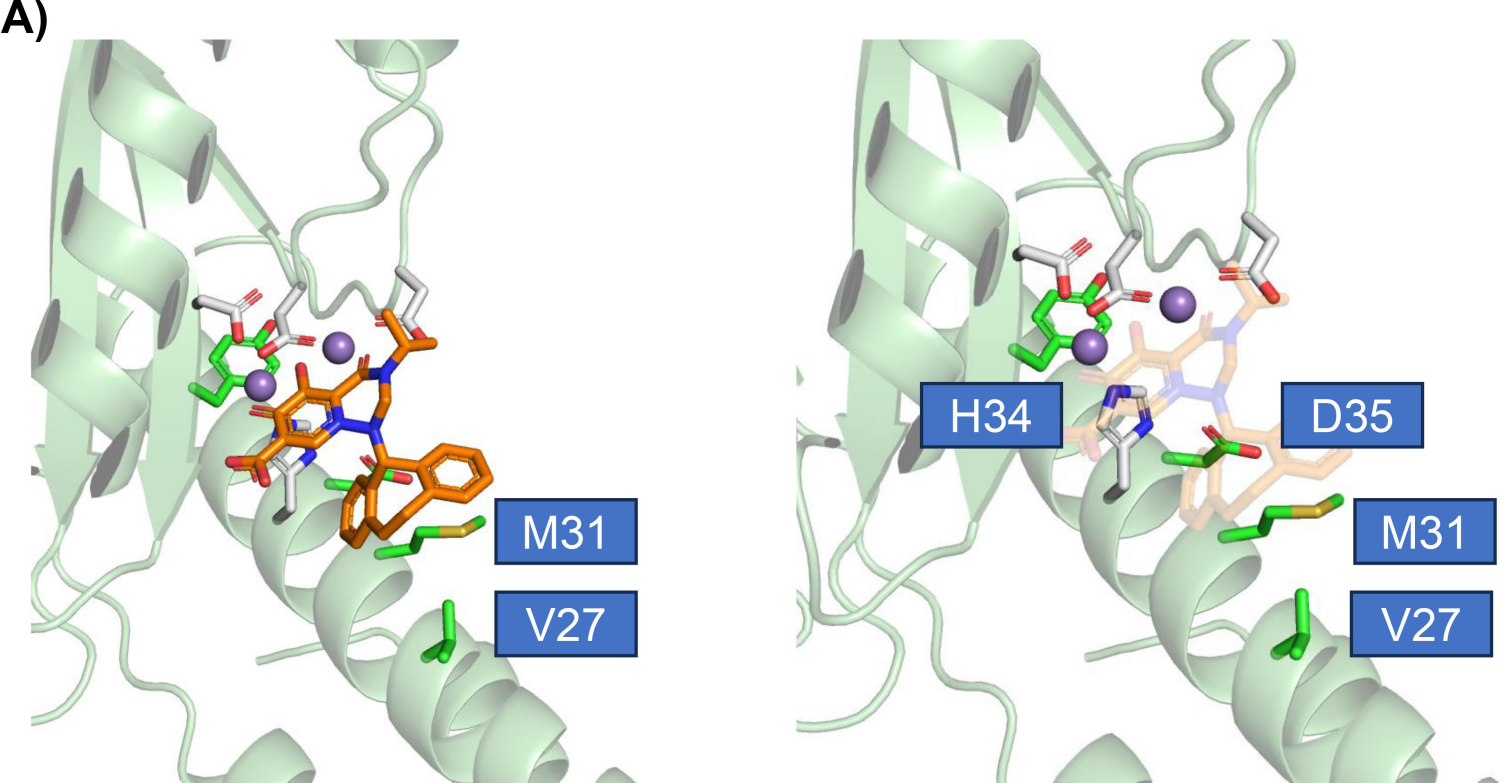
Binding model of CAPCA-1 with LACV CEN. (A) Superimposed structures of CAPCA-1 bound to the active site of the LACV CEN (PDB ID: 7PLR). Asp35, Met31, and Val27 are the amino acids identified in the CAPCA-1-resistant LACV selection assays. His34 is an amino acid that coordinates two divalent metal ions. The right image displays a faint overlay of CAPCA-1.

In a previous study, LACV with mutations in the CEN active site could not be detected during the passage of CAPCA-1, leaving it unclear whether CAPCA-1 targets the CEN of LACV (23). However, we successfully selected LACV variants with amino acid mutations in the CEN active site (Val27, Met31, Asp35, Ile47, Arg50, and Tyr93) (Fig. 4B and C). Additionally, the antiviral activity of CAPCA-1 against LACV with the V27A or D35G mutation was reduced in both Vero and SH-SY5Y cells (Fig. 4D and E; Fig. S2A and B; Table 2; Table S3). Co-crystal structure analysis with BXA and IFV CEN suggests that Lys34 and Ile38 in the IFV CEN interact with the dibenzothiepin moiety (opposite the chelate-binding site) (39). These amino acid positions correspond to Val27 and Met31 in the LACV CEN structure, where mutations were identified in this study (Fig. 4B and C) (22). Based on these findings, we hypothesize that the dihydrodibenzoannulene moiety of CAPCA-1 interacts with the Val27 and Met31 residues in the LACV CEN (Fig. 6A). Moreover, multiple substitutions at the Asp35 residue were identified in two independent experiments, including D35N, D35V, and D35G. Asp35 is adjacent to His34, an amino acid essential for metal coordination, and is highly conserved across bunyaviruses (18). This observation suggests that Asp35 is an important residue in the antiviral activity of the CEN inhibitor (Fig. 6A). Moreover, time-of-addition experiments demonstrated that CAPCA-1 inhibits viral replication. These results support the view that CAPCA-1 targets the CEN of LACV.

One limitation of the present study is related to the target validation of CAPCA-1. The CEN mutants used in this study were not recombinant viruses recovered from the cDNA clone. While no mutations were found in the L protein domain outside of CEN, several mutations were present in the Gn, NSm, and Gc proteins. To improve the accuracy of target validation, future studies should employ recombinant viruses containing only the CEN mutations to confirm whether the reduction in CAPCA-1 susceptibility is specifically attributable to those mutations.

Our study has demonstrated that CAPCA-1 exhibits anti-LACV activity *in vitro* and *in vivo*. Thus, this compound may possibly be a potential treatment option for patients with La Crosse encephalitis.

## Data Availability

The fastq files for CAPCA-1-resistant virus isolation studies (Fig. 4B) are available under the following accession numbers: DRR698768 (LACV passaged under 30 µM CAPCA-1 in experiment 1), DRR698769 (LACV passaged under 15 µM CAPCA-1 in experiment 1), DRR698770 (LACV passaged under 7.5 µM CAPCA-1 in experiment 1), DRR698771 (LACV passaged under 3.8 µM CAPCA-1 in experiment 1), DRR698772 (LACV passaged under 1.9 µM CAPCA-1 in experiment 1), DRR698773 (LACV passaged under 0.9 µM CAPCA-1 in experiment 1), DRR698774 (LACV passaged under 0 µM CAPCA-1 in experiment 1), DRR698775 (LACV passaged under 30 µM CAPCA-1 in experiment 2), DRR698776 (LACV passaged under 15 µM CAPCA-1 in experiment 2), DRR698777 (LACV passaged under 7.5 µM CAPCA-1 in experiment 2), DRR698778 (LACV passaged under 3.8 µM CAPCA-1 in experiment 2), DRR698779 (LACV passaged under 1.9 µM CAPCA-1 in experiment 2), DRR698780 (LACV passaged under 0.9 µM CAPCA-1 in experiment 2), and DRR698781 (LACV passaged under 0 µM CAPCA-1 in experiment 2). The fastq files for susceptibility studies of CEN mutants to CAPCA-1 (Fig. 4D and Table S3) are available under the following accession numbers: DRR698782 (L segment of LACV stock virus), DRR698783 (M and S segments of LACV stock virus), DRR698784 (L, M, and S segments of LACV passage control WT), DRR698785 (L segment of LACV V27A mutant), DRR698786 (M and S segments of LACV V27A mutant), DRR698787 (L segment of LACV D35G mutant), and DRR698788 (M and S segments of LACV D35G mutant).

## SUPPLEMENTAL MATERIAL

Supplemental figures aac.00186-25-s0001.pdf Fig. S1 to S5.

Supplemental material aac.00186-25-s0002.docx Tables S1 to S3; Supplemental figure legends.

ASM does not own the copyrights to Supplemental Material that may be linked to, or accessed through, an article. The authors have granted ASM a non-exclusive, world-wide license to publish the Supplemental Material files. Please contact the corresponding author directly for reuse.
