# Supplementary material for: A cap-dependent endonuclease inhibitor acts as a potent antiviral agent against La Crosse virus infection
Source: Antimicrob Agents Chemother. 2025 Jul 23;69(9):e00186-25. doi: 10.1128/aac.00186-25 (PMC12406681; doi:10.1128/aac.00186-25)

Supplementary Figure.1 Anti-LACV activity of CAPCA-1, 2, 3, 4 and 5

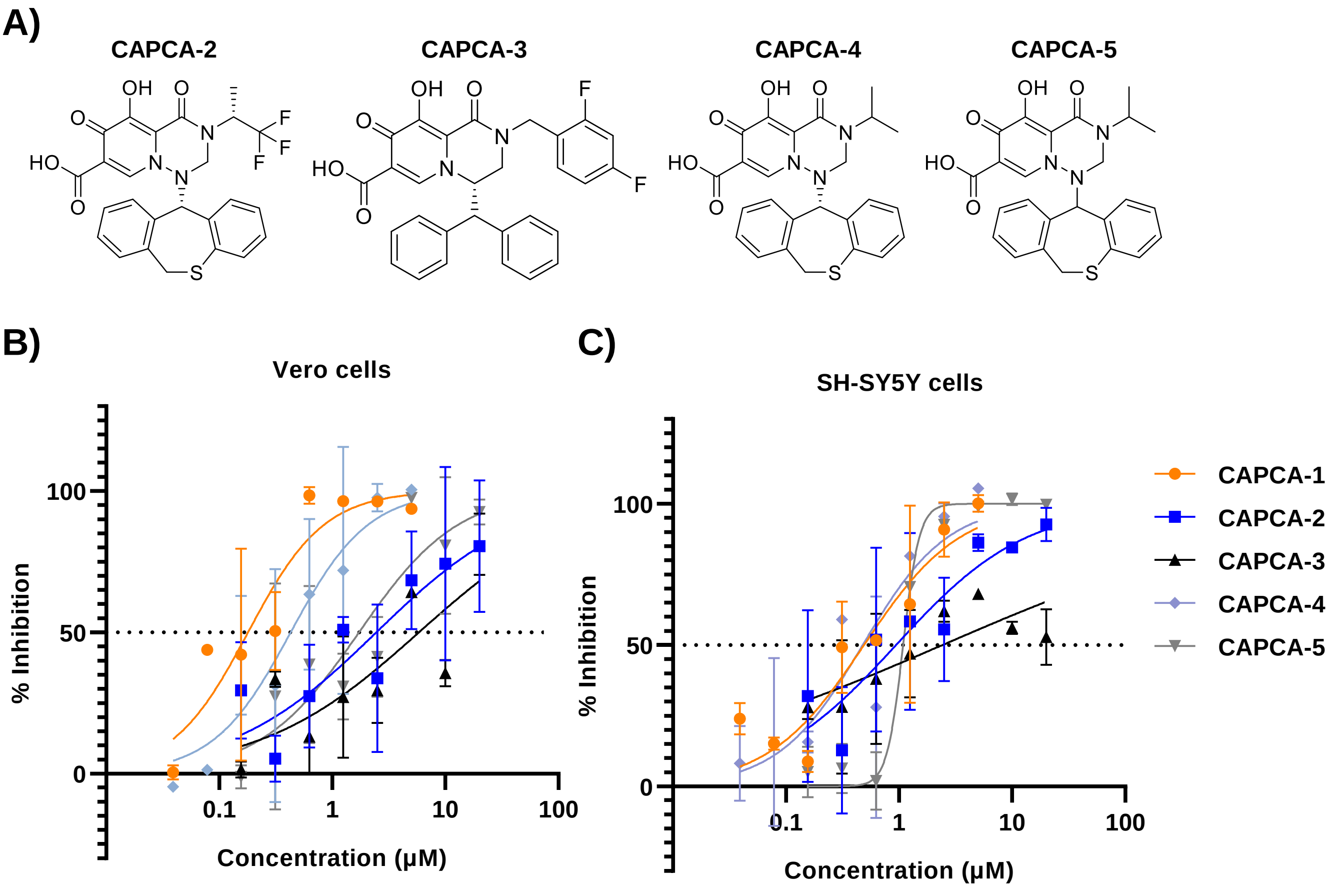

Supplementary Figure.2 *In vitro* characterization of V27A and D35G mutants

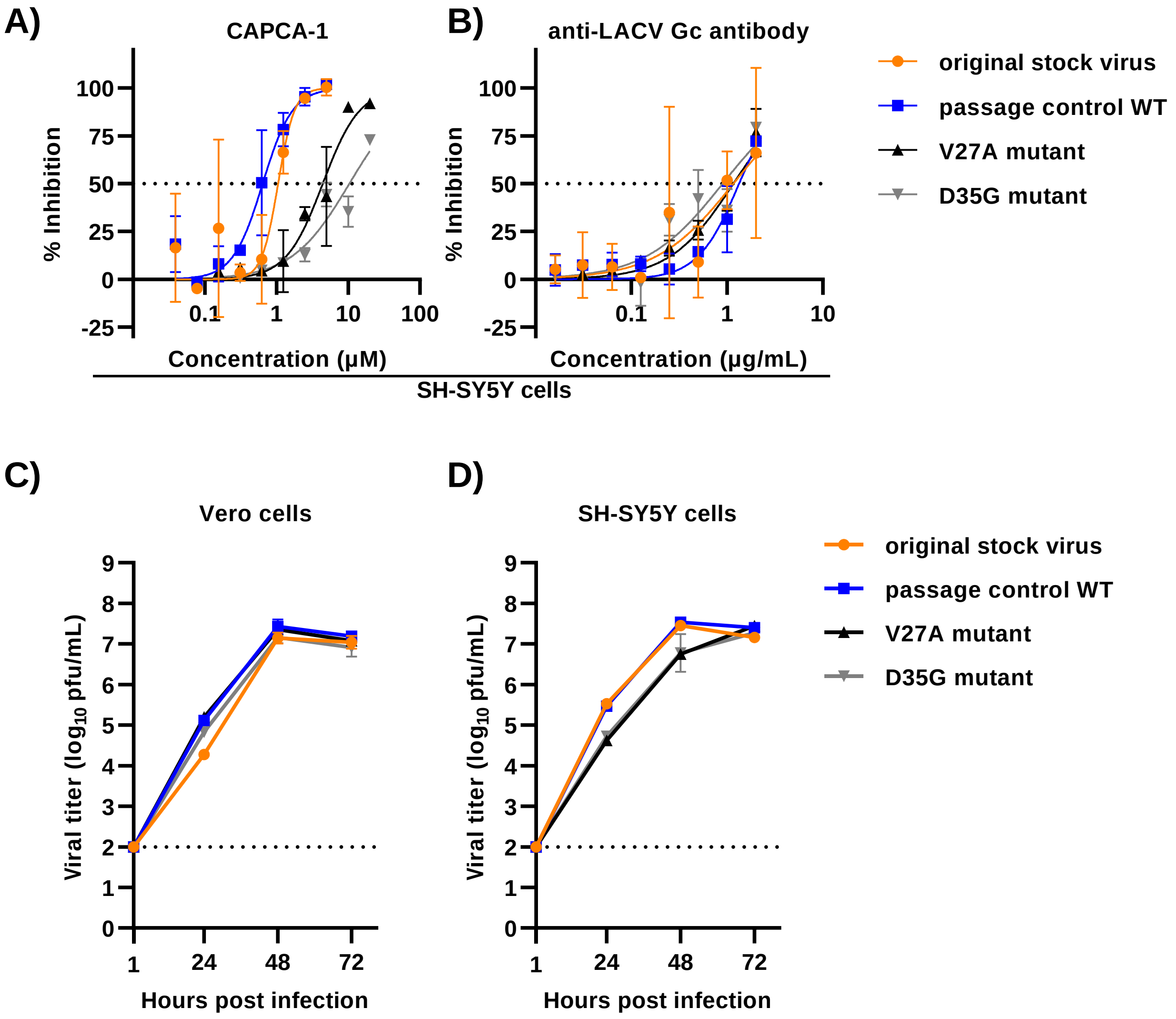

Supplementary Figure 3. Effect of CAPCA-1 administration on body weight in uninfected mice

A)

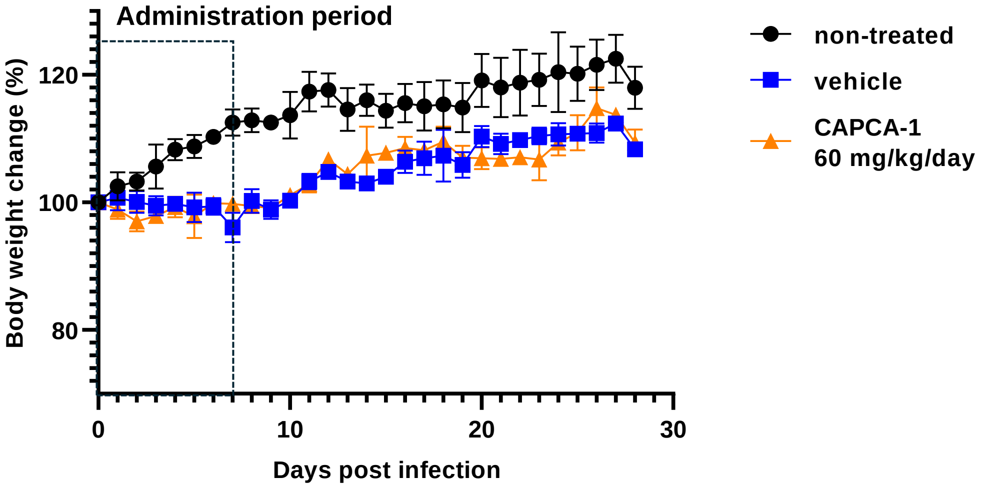

Supplementary Figure 4. *In vivo* antiviral effect of T-705 in the LACV-infected mouse model

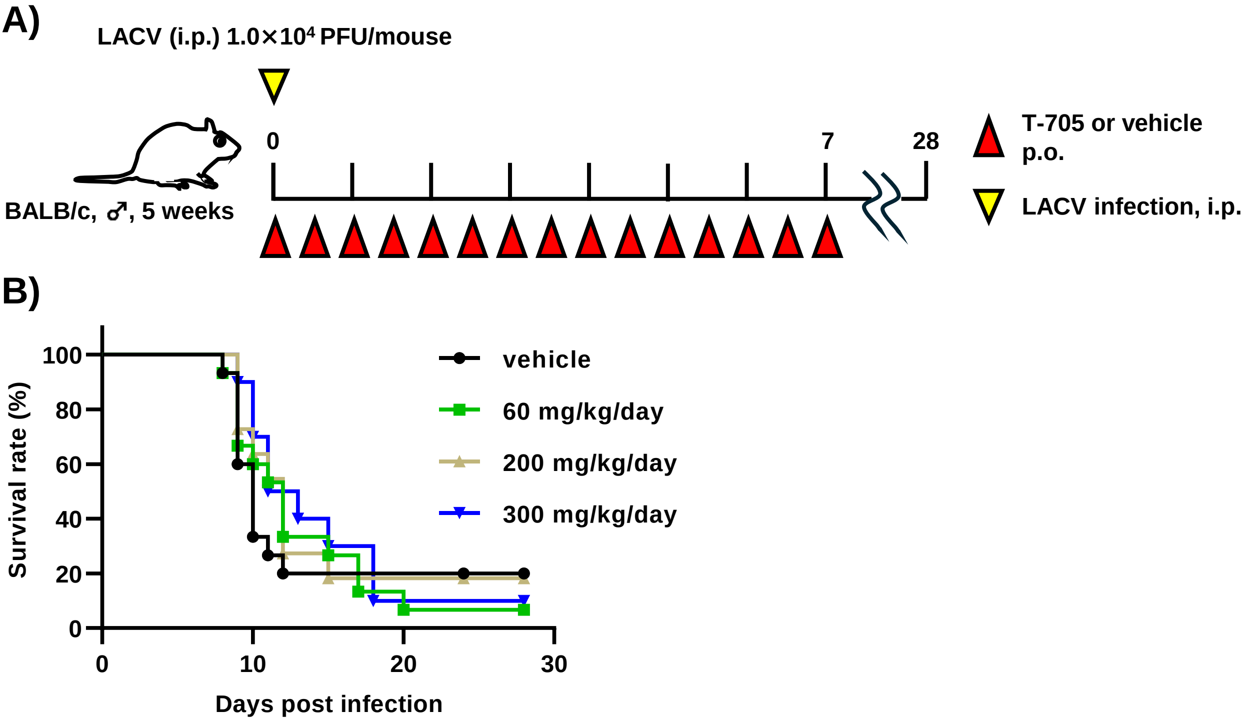

Supplementary Figure 5. *In vivo* antiviral effect of CAPCA-1 in LACV-infected mouse via footpad inoculation

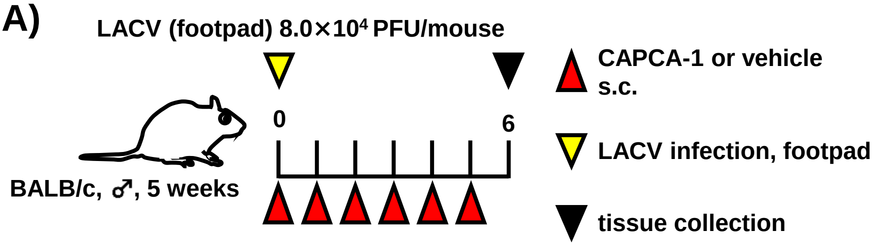

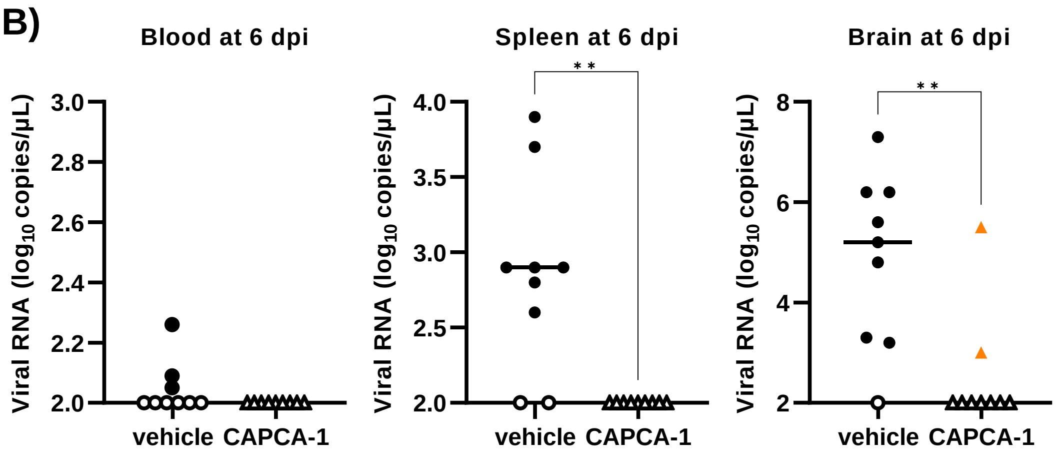

Supplement: Supplemental figures — Fig. S1 to S5. [file aac.00186-25-s0001.pdf]
