# Supplementary material for: A cap-dependent endonuclease inhibitor acts as a potent antiviral agent against La Crosse virus infection
Source: Antimicrob Agents Chemother. 2025 Jul 23;69(9):e00186-25. doi: 10.1128/aac.00186-25 (PMC12406681; doi:10.1128/aac.00186-25)
Supplement: Supplemental material — Tables S1 to S3; Supplemental figure legends. [file aac.00186-25-s0002.docx]

**Supplementary Table 1**

Sequence of primers and probes for the NGS analysis**.**

| target | Forward primer (5′ → 3′) | Reverse primer (5′ → 3′) |
| --- | --- | --- |
| L gene 1 | AGTAGTGTACTCCTATCTACAAAACTTAC | TATTTTCTATTCTGTTCCGCAAGTGG |
| L gene 2 | CCAGCAATGTTAAGGACTATATAGC | CGGAATATCACAGATTCCATATAATC |
| L gene 3 | GAGTCAGTAGTCAACCAAATAGCTG | AGTAGTGTGCTCCTATCTACAAATTTA |
| M gene 1 | AGTAGTGTACTACCAAGTATAGATAACG | GGATATTCAATTTCCCAACATTTGG |
| M gene 2 | GATGACTTGATTGCATACACAAATAAG | AGTAGTGTGCTACCAAGTATAAAATAATG |
| S gene | AGTAGTGTACTCCACTTGAATACTTTG | AGTAGTGTGCTCCACTGAATACA |

**Supplementary Table 2**

Amino acid substitutions observed in the cording region of original stock virus, passage control WT, V27A mutant and D35G mutant (top, L segment; bottom, M segment). The amino acids listed in the Table represent substitutions with a mutation frequency of 20% or higher. Reference sequences used were: L segment – NC_077810 and M segment – NC_077809. No amino acid mutations were observed in the coding region of the S segment.

|  | L segment | | | |
| --- | --- | --- | --- | --- |
|  | CEN domein | |  | |
|  | V27A | D35G | K1440R | T1689A |
| Original stock virus |  |  |  |  |
| Passage control WT |  |  | 60 | 36 |
| V27A mutant | 100 |  |  |  |
| D35G mutant |  | 100 |  |  |

| M segment | | | | | | | | |
| --- | --- | --- | --- | --- | --- | --- | --- | --- |
|  | Gn | NSm | | Gc | | | | |
|  | P307H | L359P | G411R | H602N | W618R | N675D | D684N | K698E |
| Original stock virus |  | 53 | 100 |  | 68 |  | 100 | 24 |
| Passage control WT | 90 | 97 | 100 | 95 | 98 |  | 100 |  |
| V27A mutant |  | 100 | 100 |  | 100 | 93 | 100 |  |
| D35G mutant |  | 100 | 100 |  | 100 | 91 | 100 |  |

**Supplementary Table 3**

Antiviral activity of CAPCA-1 or anti-LACV Gc monoclonal antibody (clone 8C2.2) against the original stock virus, passage control WT, and the V27A and D35G mutants in SH-SY5Y cells. EC_50_ (µM, µg/mL) is the concentration of the antivirals that inhibited LACV-induced CPE by 50% relative to the non-treated control. The fold change refers to the ratio of the EC_50_ value for each virus to that of the original stock virus or passage control WT. Data are represented as the mean ± SD.

|  | Original stock virus | Passage control WT | V27A mutant | D35G mutant |
| --- | --- | --- | --- | --- |
| CAPCA-1 EC_50_ (µM) | 0.95±0.16 | 0.65±0.05 | 4.63±0.80 | 12.89±2.22 |
| fold change (/original stock) | 1.00 | 0.68 | 4.87 | 13.57 |
| fold change (/passage control) | 1.46 | 1.00 | 7.12 | 19.83 |
| Anti-LACV Gc monoclonal antibody EC_50_ (µg/mL) | 0.99±0.35 | 1.12±0.18 | 1.28±0.18 | 1.21±0.39 |
| fold change (/original stock) | 1.00 | 1.13 | 1.29 | 1.22 |
| fold change (/passage control) | 0.88 | 1.00 | 1.14 | 1.08 |

**Figure legends**

**Supplementary Figure 1. Anti-LACV activity of CAPCA-1, 2, 3, 4 and 5**

**(A**) Chemical structure of CAPCA-2, 3, 4 and 5. **(B, C)** CPE-based antiviral activity assay. Vero (B) or SH-SY5Y (C) cells were treated with serially diluted compounds and infected with LACV. The inhibition rate was assessed using an MTT assay. The dotted lines indicate 50% inhibition. Data are represented as the mean ± SD. CAPCA-1, 2, 3, 4 and 5.are represented by orange, blue, black, purple, and gray lines, respectively. These data are representative of three independent experiments performed in duplicate.

**Supplementary Figure 2. *In vitro* characterization of V27A and D35G mutants**

**(A, B)** CPE-based antiviral activity assay. SH-SY5Y cells were treated with CAPCA-1 (A) or anti-LACV Gc antibody (B) and infected with LACV. The inhibition rate was assessed using an MTT assay at 3 dpi. The dotted lines indicate 50% inhibition. These data are representative of more than three independent experiments performed in duplicate. **(C, D)** Growth curves of original stock virus, passage control WT, V27A mutant or D35G mutant in Vero cells (C) and SH-SY5Y cells (D). The cells were infected with LACV at a MOI of 0.01. Viral titers in the supernatants were quantified using a plaque assay at 1, 24, 48 and 72 hpi. The dotted lines indicate limit of detection. These data are representative of two independent experiments performed in triplicate. Data are represented as the mean ± SD (A-D). The original stock virus, passage control WT, V27A mutant and D35G mutant are represented by orange, blue, black, and gray lines, respectively (A-D).

**Supplementary Figure 3. Effect of CAPCA-1 administration on body weight in uninfected mice**
**(A)** Body weight changes of non-treated, vehicle or CAPCA-1 treated mice (*n* = 3 each group). Body weights were monitored daily in uninfected mice treated subcutaneously with CAPCA-1 or vehicle control for 7 consecutive days. The dotted lines indicate the administration period. Data are represented as the mean ± SD.

**Supplementary Figure 4. *In vivo* antiviral effect of T-705 in the LACV-infected mouse model**

**(A)** Schematic representation of the experimental design of the prophylactic administration model. Mice were administered vehicle or T-705 4 h before infection and subsequently intraperitoneally infected with LACV (1.0 × 10⁴ PFU/mouse). Treatment was continued until 7 dpi (b.i.d., 60, 200 or 300 mg/kg/day). i.p., intraperitoneally; p.o., orally. **(B)** Survival rates in LACV-infected mice treated with the vehicle (*n* = 15) or T-705 (60 mg/kg/day; *n* = 15, 200 mg/kg/day; *n* = 11, 300 mg/kg/day; *n* = 10). Statistical analysis was performed using the log-rank (Mantel–Cox) test. *p-*values from the survival analysis were adjusted using the Bonferroni correction, and *p*-value of less than 0.0167 (0.05/3) was considered statistically significant.

**Supplementary Figure 5. *In vivo* antiviral effect of CAPCA-1 in LACV-infected mouse via footpad inoculation**

**(A)** Schematic representation of the experimental design of the prophylactic administration model. Mice were administered vehicle or CAPCA-1 4 h before infection and subsequently infected with LACV (8.0 × 10⁴ PFU/mouse) via footpad. Treatment was continued until 5 dpi (q.d., 60 mg/kg/day). s. c., subcutaneously. **(B)** Viral RNA in the blood (left), spleen (middle), and brain (right) in LACV-infected mice treated with vehicle or CAPCA-1 (60 mg/kg) at 6 dpi. Each dot indicates individual mice. The white circle or triangle indicates below the lower limit of quantification. The bar indicates the median. Statistical analysis was performed by the Mann–Whitney U test. ***p* < 0.01.
